# Dietary Patterns Derived from Reduced Rank Regression Are Associated with the 5-Year Occurrence of Metabolic Syndrome: Aichi Workers’ Cohort Study

**DOI:** 10.3390/nu14153019

**Published:** 2022-07-22

**Authors:** Yuanying Li, Hiroshi Yatsuya, Chaochen Wang, Mayu Uemura, Masaaki Matsunaga, Yupeng He, Maythet Khine, Atsuhiko Ota

**Affiliations:** 1 Department of Public Health, Fujita Health University School of Medicine, Toyoake 470-1192, Japan; mm-223@fujita-hu.ac.jp (M.M.); yupeng.he@fujita-hu.ac.jp (Y.H.); may-thet@fujita-hu.ac.jp (M.K.); ohtaa@fujita-hu.ac.jp (A.O.); 2 Department of Public Health and Health Systems, Nagoya University Graduate School of Medicine, Nagoya 466-8550, Japan; h828@med.nagoya-u.ac.jp (H.Y.); mayu-u@fmu.ac.jp (M.U.); 3 Japan Value, Evidence and Outcomes, Japan Drug Development and Medical Affairs, Eli Lilly Japan K.K., Kobe 651-0086, Japan; wang_chaochen@lilly.com; 4 Department of Epidemiology, School of Medicine, Fukushima Medical University, Fukushima 960-1295, Japan

**Keywords:** dietary pattern, nutrients, metabolic syndrome, prospective, worker, reduced rank regression

## Abstract

The aim of the present study was to derive dietary patterns to explain variation in a set of nutrient intakes or in the measurements of waist circumference (WC) and fasting blood glucose (FBG) using reduced rank regression (RRR) and to prospectively investigate these patterns in relation to the risk of developing metabolic syndrome (MetS) and its components during the follow-up. The study participants were comprised of 2944 government employees aged 30–59 years without MetS. RRR was applied with 38 food groups as predictors and with two sets of response variables. The first set included intake of putatively beneficial nutrients, and the first factor retained was named the Healthy Dietary Pattern (HDP). The second one included baseline WC and FBG, and the first factor was named the Unhealthy Dietary Pattern (UHDP). Multivariable Cox proportional hazard model was used to estimate hazard ratio and 95% confidence intervals with adjustments for age, sex, total energy consumption and other potential confounders. During the 5-year median follow-up, we ascertained 374 cases of MetS. The HDP score was inversely associated with the incidence of MetS (*p*-trend = 0.009) and hypertension (*p*-trend = 0.002) and marginally significantly associated with elevated triglyceride and decreased high-density lipoprotein cholesterol (*p*-trend = 0.08). The UHDP score was linearly positively associated with the incidence of MetS and all its components (all *p*-trend < 0.05). Both the HDP and UHDP predicted the development of MetS and its components.

## 1. Introduction

Metabolic syndrome (MetS) is a state of increased body adiposity, blood pressure, blood glucose and unfavorable serum lipid levels. The estimated prevalence of MetS in adults varies between countries, from at least 10% up to 30–40% [1], representing a significant individual and public health burden, and it is a precursor of cardiovascular disease (CVD) [2] and dementia [3].

Diet quality, one of the modifiable lifestyle factors, plays a critical role in the maintenance of metabolic health. Many studies have linked food or dietary pattern with the prevalence of MetS [4,5,6,7,8,9]. However, few prospective studies have evaluated this association.

Two studies conducted in the United States of America (USA) applied exploratory or cluster analyses, which are effective at capturing the correlational structure of foods, and found no associations of Healthy/Prudent dietary patterns with the development of MetS [10,11]. Other prospective studies based on the populations of the USA [12,13], Spain [14] and France [15] observed that people adherent to the Mediterranean diet [13,14] or Nutritional Guidelines [12,15] experienced a lower risk of MetS incidence.

Considering that food and dietary habits are highly culture-specific, more studies in Asian populations are warranted; only two studies based on Koreans reported that a higher Healthy dietary score derived from factor analysis [16] was negatively, while adherence to Unhealthful plant-based diet [17] was positively, related to MetS incidence and certain components.

Reduced rank regression (RRR) is an alternative dimension-reduction technique that derives dietary patterns which explain maximum variation of a set of predetermined response variables, typically hypothesized mediators of a disease [18]. Thus, it is also expected to provide insight into the pathophysiological pathways that link dietary patterns to disease [19]. In the present study, we attempted to extract two dietary patterns, each using different sets of response variables. One included the intake of beneficial nutrients that are reported to be inversely associated with MetS risk. The other response variables were determined according to the finding of the analysis using the first dietary pattern in relation to each MetS component. Namely, we used baseline values of waist circumference (WC) and fasting blood glucose (FBG) as response variables, since no association was found for the first dietary pattern with the onset of abdominal obesity and high FBG in the present study.

A variety of nutrients have been linked to MetS or its components. Higher intakes of total fiber [8,20,21], vitamin C (VC) [22], magnesium (Mg) [23,24], potassium (K) [25], vitamin A or β-carotene [26], vitamin E (VE) [27] and omega-3 polyunsaturated (n3) fatty acids [28,29,30] and consuming relatively few carbohydrates [31,32] are inversely associated with MetS or its components in observational studies.

Thus, in the present study, we aimed to derive two dietary patterns using RRR and prospectively explore their association with the incidence of overall MetS and each component during a 5-year follow-up in a Japanese cohort of middle-aged government employees.

## 2. Materials and Methods

### 2.1. Study Subjects

The Aichi Workers’ Cohort Study is an ongoing prospective cohort study initiated in 1997 to elucidate the causative factors of diabetes and CVD. The participants comprise workers and retirees among civil servants in Aichi prefecture in central Japan. The target subjects of the present study were working employees who provided written informed consent (*n* = 5061) to use the data collected from the fourth baseline survey conducted in 2013 and the provision of annual health check-up data from 2013 to 2018.

In the baseline year of 2013, 5610 and 4836 participants completed the questionnaire surveys and health check-ups, respectively. The present study was restricted to participants aged 30 to 59 years (*n* = 4193), and we excluded those with a self-reported history of CVD (*n* = 51) and those with any missing values for baseline data on blood pressure and WC (*n* = 189), measures of blood biomarkers (*n* = 291), medical histories (*n* = 207) and lifestyles (*n* = 24). We further excluded participants with MetS at baseline (*n* = 261), those without follow-up data (*n* = 35) and those reporting implausible energy intakes (*n* = 191), defined using the sex-specific 2.5th and 97.5th percentiles (<1084 or >3957 kcal/day for men and <890 or >3606 kcal/day for women), leaving 2944 (2103 men and 841 women) participants in the final analysis for MetS incidence. When analyzing the incidence of individual MetS components, the baseline prevalence of the respective risk factor was excluded, thus resulting in varied numbers for each subset population.

### 2.2. Questionnaire (Food Frequency Questionnaire (FFQ) and Covariates)

The paper-based questionnaires were distributed through the workplace and the participants were asked to recall and self-report their usual food intake during a preceding year using the FFQ, which included 172 food and beverage items. There were nine frequency categories and three portion-size categories. For most foods, the consumption frequency was ranging from “lower than once/month” to “more than seven times/day”. A standard portion size was specified for each food item, and the respondents were asked to estimate their usual portion sizes for each food using three options (less than half, standard, and more than one and a half times).

The validity and reproducibility were established among middle-aged and elderly Japanese adults. The median (range) values of the deattenuated energy-adjusted Spearman coefficients of the correlation between the FFQ responses and 12-day weighted food records for the intakes of 29 food groups were 0.46 (0.22–0.75) for men and 0.48 (0.06–0.80) for women [33], while those for the intakes of 53 nutrients were 0.50 (0.01–0.82) for men and 0.43 (0.14–0.74) for women. The corresponding values for the reproducibility based on two FFQs were 0.55 (0.42–0.84) for men and 0.54 (0.35–0.80) for women for 53 nutrients, and 0.54 (0.39–0.79) for men and 0.57 (0.40–0.83) for women for 29 food groups [34].

Baseline information on lifestyles and disease history related to CVD, hypertension, dyslipidemia and diabetes (yes or no) was self-reported in the questionnaire. Smoking status was classified into never, former, current at <20 cigarettes/day and current at ≥20 cigarettes/day. Daily ethanol intake (g/day) and daily total physical activity (metabolic equivalents of task (MET)-hours/day) [35] were grouped into sex-specific quartiles. Educational background was dichotomized into lower than university education and others. Type of work was self-reported as clerical or non-clerical. Changes in eating habits were assessed as no change, changed within 1 year, changed in the past 1–2 years and changed in the past 3–5 years. Body mass index (BMI) was calculated as weight in kilogram divided by squared height in meters.

### 2.3. Definition of MetS and Follow-Up

MetS was defined according to the common criteria for the clinical diagnosis of MetS with the WC cutoff for the Asian population [36]. A participant was deemed to have MetS when three or more of the following criteria were reached:

(1) Abdominal obesity: WC ≥ 90 cm in men and WC ≥ 80 cm in women;
(2) Hypertension: systolic blood pressure (SBP) ≥ 130 mmHg and/or diastolic blood pressure (DBP) ≥ 85 mmHg, and/or the use of antihypertensive medications;
(3) High triglycerides (TG), ≥150 mg/dL and/or the use of medication for dyslipidemia;
(4) Low high-density lipoprotein cholesterol (HDLC), <40 mg/dL in men and HDLC < 50 mg/dL in women, and/or the use of medications for dyslipidemia;
(5) High FBG, ≥100 mg/dL and/or the use of hyperglycemic agents.

The participants were followed up with until the incidence of MetS or censoring. The person-years of the follow-up were calculated from the baseline to the year when the MetS criteria were first met (i.e., incidence) or to the censoring (the last year when the health check-up data were confirmed). Likewise, the person-years of the individual component of MetS were calculated from baseline to the year when it first occurred or, otherwise, to the last data observed.

### 2.4. Statistical Analysis

The daily intakes of most foods were calculated by multiplying the daily consumption frequency and the standard portion size by the individual’s usual portion size. The total daily energy intake and intake of each nutrient were estimated according to the Japanese standard tables of food composition [37].

Some beverages were excluded from the analysis, such as herbal teas that generally have no energy and juices that only 10–20% of the participants regularly drank more than 1–2 times/week. Spices and seasonings were not considered food materials and, therefore, were excluded. Based on the similarity in nutritional content or culinary usage, the remaining foods were aggregated into 38 food groups and modeled as predictors of RRR. We only focused on the first pattern derived by RRR for the subsequent analyses, as this pattern covered the larger proportion of explained variance in the response variables. The Healthy Dietary Pattern (HDP) was derived by explaining the variation in eight nutrients, which were reportedly favorable for preventing MetS or its components based on observational studies, including the intakes of total fiber, β-carotene, VC, VE, K, Mg and n3 fatty acids, and a higher ratio of protein to carbohydrates. Another dietary pattern, the Unhealthy Dietary Pattern (UHDP), was extracted by setting the baseline WC and FBG as response variables, as the preceding HDP was not associated with the incidence of abdominal obesity and elevated FBG in the present study. Thus, we attempted a post hoc procedure to explore which combinations of foods had a significant influence on these two components.

Before the RRR procedure, all the selected nutrient intakes were divided by the total energy intake [38]. All the predictors and response variables were logarithmically transformed. Pearson’s correlation coefficients were used to assess the relationships between the dietary pattern scores and food groups, as well as response variables.

The cutoff values for the quartiles of dietary score were determined by averaging the sex-specific quartile cutoff values. The differences in the means for continuous variables and proportions for categorized variables across quartiles of dietary pattern scores were tested using a generalized linear model procedure.

Cox proportional-hazard regression was used to assess the association between the quartiles of dietary scores and incidence of MetS, as well as its components. The hazard ratio (HR) and 95% confidence intervals (CIs) were calculated using the first quartiles of the factor score as the reference. We construed two models. Model 1 was adjusted for baseline age (continuous), sex and total energy intake (continuous). Model 2 was additionally adjusted for smoking status (never, former, current at <20 cigarettes/day and current at ≥20 cigarettes/day), sex-specific quartiles of alcohol intake (g/day) and total physical activity (MET-hours/day), educational background (lower than university, or others), occupation (clerical, or non-clerical), change in eating habits (no change, changed within 1 year, changed in the past 1–2 years and changed in the past 3–5 years) and the use of medication for dyslipidemia (yes or no), antihypertension (yes or no) and hyperglycemia (yes or no).

The trends across quartiles were tested using dietary pattern scores modeled as the median value of quartile, with which the effect modification by sex was tested by including multiplicative term of sex.

In order to account for multiplicity of testing related to multiple outcomes (i.e., six), we adjusted probability to detect the statistical significance using the Holm-Bonferroni procedure [39].

All the data analyses were conducted using the SAS software version 9.4 (SAS Institute Inc., Cary, NC, USA), with two-sided *p*-values < 0.05 considered to be significant.

## 3. Results

The factor loadings for each dietary pattern are presented in Table 1. HDP was characterized by a higher intake of vegetables, fruits and soy products; a moderate intake of fish, dairy products, pickles and millet; and a lower intake of rice. This pattern explained 35.3% of the variations in nutrient intakes and 13.1% of those in food intake. The HDP score was positively significantly (all *p* < 0.001) associated with response variables, with Pearson correlation coefficients of 0.75 (total fiber), 0.68 (VC), 0.66 (VE), 0.63 (K), 0.58 (Mg), 0.57 (β-carotene), 0.56 (n3 fatty acids), 0.40 (protein), 0.34 (ratio of protein to carbohydrates) and 0.16 (carbohydrates).

In contrast to the HDP, the UHDP is typified by a diet lower in vegetables, bread, sweeteners, millet, mushrooms and fruit and higher in pickles, rice, noodles, beef, liver, seafood other than fish and processed meats. It explains 4.1% of the variation in the response variables and 3.8% of that in food intake. The Pearson correlations between the UHDP score and WC and FBG were 0.25 and 0.15, respectively, with *p* values < 0.001 (Table 1).

The mean (standard deviation) baseline age was 45.5 (8.1) years, with men comprising 86% of the participants. Participants in the higher quartile of HDP were more likely to be female, have a lower than university education, be never smokers, be physically active and consume more total energy, but tended to have lower BMI, alcohol consumption, SBP, DBP, HDLC and TG (Table 2).

There was no difference detected for the proportion of education, job type, change in eating habits, total physical activity, and medication use for hypertension and hyperglycemia across the quartiles of UHDP, whereas the proportion of male subjects, former and current smokers, and all remaining continuous variables tended to increase along with UHDP quartile (Table 3).

During a 5-year median follow-up, 374 participants (men: 286; women: 88) developed MetS. Compared to those in the lowest quartiles, participants in the third and fourth quartiles of HDP were 35% and 40% less likely to develop MetS independent of age, sex and total energy intake with HRs (95% CI) of 0.65 (0.49–0.88) and 0.60 (0.44–0.82), respectively. After additionally adjusting for other lifestyle, education and occupational background, as well as the medication uses, the corresponding HRs (95% CI) were slightly attenuated, at 0.68 (0.50–0.91) and 0.65 (0.48–0.90), with a negative monotonic trend (*p* = 0.002). The fully adjusted HRs (95% CI) for hypertension were 0.81 (0.67–0.97), 0.82 (0.68–1.00) and 0.71 (0.57–0.88) from the second to fourth quartiles of HDP (*p*-trend = 0.002). The quartiles of the HDP scores were marginally significantly inversely associated with elevated TG and low HDLP (*p*-trend = 0.08) (Table 4).

The UHDP score was linearly positively associated with the incidence of MetS and all the individual components (all *p*-trend < 0.05). The fully adjusted HRs (95% CIs) across the second, third and fourth quartiles were 1.48 (1.01–2.16), 2.01 (1.40–2.88) and 2.51 (1.77–3.54) for MetS incidence; 1.37 (1.00–1.86), 1.61 (1.17–2.21) and 1.93 (1.42–2.62) for abdominal obesity; and 1.30 (0.96–1.76), 1.31 (0.98–1.76) and 1.46 (1.09–1.97) for elevated FBG, respectively. The corresponding estimates were 1.42 (1.07–1.90), 1.37 (1.03–1.83) and 1.60 (1.21–2.12) for elevated TG; 1.58 (1.04–2.40), 2.06 (1.39–3.06) and 1.94 (1.30–2.88) for low HDLC; and 1.13 (0.90–1.41), 1.29 (1.04–1.60) and 1.39 (1.12–1.72) for hypertension, respectively (Table 5).

No significant effect modification by sex was observed for the association of dietary patterns and MetS incidence or its components (all *p*-interactions > 0.10), except for UHDP quartiles with abdominal obesity (*p*-interaction = 0.01, model 2). The fully adjusted HRs (95% CIs) of abdominal obesity across the second, third and fourth quartiles were 1.31 (0.92–1.88), 1.26 (0.85–1.89) and 1.31 (0.83–2.07) for women (*p*-trend = 0.18) and 1.67 (0.88–3.16), 2.47 (1.35–4.52) and 3.04 (1.71–5.42) for men (*p*-trend < 0.0001).

The Holm-Bonferroni method implied that *p*-values for the quartiles of UHDP equal or larger than 0.01 might simply indicate statistically non-significant findings. However, the number of outcomes and their respective criteria were not determined arbitrarily in the present study. Moreover, each hypothesis has both biological plausibility and empirical support from previous human studies. Thus, the dose-response associations detected in the present study with empirical *p*-values (0.05) would like to reflect the actual relationships in nature, not just a finding by chance [40].

## 4. Discussion

Dietary patterns beneficial to CVD and general health share several key characteristics, which include more minimally processed foods such as vegetables, fruit, legumes, nuts, whole grains, yogurt, vegetable oils and seafood, and less red meats, processed meats, refined grains, starches and added sugars [41]. The HDP and UHDP extracted in the present study conceptually reflected a combination of these food items and, as expected, intake of HDP and UHDP was negatively correlated (Pearson correlation = −0.39).

The overall MetS incidence was reduced by 35% in the participants with the highest quartile of HDP intake, and it was increased by 150% in the highest quartile of UHDP intake compared with the lowest quartile. The finding related to the UHDP was more striking than that for the HDP, which seems reasonable, as the HDP was derived by explaining hypothesized beneficial nutrient intakes while the UHDP combined food in a way that explained the baseline WC and FBG, which are expected to be more closely involved in the pathogenesis of MetS [42,43].

To the best of our knowledge, only two Asian studies have generated Healthy and Unhealthy dietary patterns and prospectively examined the association between diet quality and MetS risk. Similar to our study, the Korean Genome Epidemiology study [16] found that factor analysis derived a Healthy dietary pattern, characterized by the high intake of green/yellow vegetables, seaweed, fish/seafood and red/processed meat and a low intake of refined white rice, was linearly associated with the 6-year incidence of MetS, abdominal obesity, elevated FBG and low HDLC, with a significant 20% to 40% risk reduction observed for the fourth and fifth quintiles compared with the lowest one. The major foods that emerged in the Unhealthy dietary pattern were analogous to the UHDP of the present study, loaded with refined white rice, red/processed meat, noodles and sweetened carbonated beverages, and lacking mixed grain rice. However, this pattern only predicted abdominal obesity [16]. Our findings on the UHDP were partially in agreement with that from the South Korean prospective cohort study [17] where individuals in the highest versus lowest quintile of Unhealthful plant-based diet score, featuring a high intake of refined carbohydrates, potatoes, sugars and salty food groups but low intake of whole-grains, vegetables, fruits, nuts, legumes and any animal foods or products, had a 50% higher risk of 8-year incidence of MetS and a 24% to 46% higher risk of abdominal obesity, high TG, low HDLC and hypertension. Regarding the elevated FBG, the risk decreased in the participants greatly adhering to the overall (Healthful and Unhealthful) plant-based diet but appeared to be flat for the Healthful plant-based diet [17].

There was another study based on a community population with USA derived Prudent and Western dietary patterns using factor analysis, and it has linked these with the development of MetS [10]. Similar to our findings, the Western dietary pattern in the ARIC study—which presented a higher intake of refined grain bread, cereal, rice and pasta, processed meat, fried foods, red meat, eggs and refined grain desserts, and a lower intake of yogurt—was positively associated with the 9-year incidence of MetS [10], with a 12% to 18% increased risk appearing across the second to fifth quintiles. There was no association found for the Prudent dietary pattern of consuming a higher amount of vegetables, fruit, fish and seafood, whole grains and legumes.

The discrepancy between the results of the studies may be due to the different dietary scores and varied prevalence of risk factors among the study populations. Moreover, the relatively consistent findings on the risk factors of MetS in the present study might be attributed to the food combination not only reflecting dietary habits specific to the study population but also nutritional and biological pathways related to MetS.

Our results for the HDP are comparable to the findings from previous Western countries’ studies that relied on a hypothesis-oriented method. The SUN prospective cohort study of Spanish populations [14] and the Framingham Offspring Cohort study of American populations [13] reported that adherence to the Mediterranean diet was inversely associated with MetS incidence. A French cohort also observed that people complying with French nutritional guidelines, which recommend the daily consumption of organic, seasonal and locally produced foods, complete starchy foods, walnut and olive oils, dairy products, and fish twice a week, were less likely to develop MetS during the 6-year follow-up [15].

Hypertension in the present study was the most prevalent risk factor of MetS, and HDP’s inverse association with hypertension seems to have largely contributed to reducing MetS incidence. The previous randomized controlled trials (RCT) demonstrated that higher intakes of dietary fiber [44], K [45], VC [46] and Mg [47] prevented hypertension. In prospective studies, greater consumption of dietary fiber, K and Mg [48] has been observed to prevent hypertension [49]. As for specific food groups, a recent meta-analysis of prospective studies demonstrated that whole grains, fruits, nuts and dairy had negative dose-response associations with the risk of hypertension, whereas red and processed meat had a positive association [50]. However, it is more likely to be the synergistic effect of the entire diet than the individual foods or nutrients protecting against hypertension, as suggested by a recent meta-analysis of controlled intervention trials [51] finding that the Dietary Approach to Stop Hypertension (DASH) diet and Lacto-Ovo Vegetarian diet, as well as Nordic and Mediterranean diets, proved to be more effective than a vegan diet or high-fruit-and-vegetable diets and high-fiber diets.

Studies of RCT [52,53] demonstrated that a high intake of fish improved blood lipid profiles, including TG and HDLC. In addition, VE supplementation resulted in statistically significant improvements in TG [54]. On the other hand, observational studies have reported detrimental effects from a high-carbohydrate diet on HDLC and TG levels in Korean [32,55] and U.S. women [32] but not in U.S. men [32]. Total red meat intake showed a significant positive association with TG and HDLC in a 13-year prospective study of a healthy Iranian population [56]. In addition, meta-analysis of RCT [57,58] and intervention trials [59] reported that substituting red meat with high-quality plant protein [58] or consumption of plant protein rather than animal protein [59], led to more favorable changes in blood lipids and lipoproteins, including TG and HDLC. However, vegetarian diets did not significantly affect blood TG concentrations, but effectively lowered the cholesterol profile including HDLC [57]. Thus, the linear association observed for TG and HDLC may be largely attributed to the plant protein, fish and red meat, as well as rice and noodles as the main sources of carbohydrates in the present study.

Elevated FBG and abdominal obesity would more closely reflect the existence of insulin resistance and visceral obesity, which are considered to be leading suggestive pathophysiologies of MetS [42,43]. The UHDP intake consistently predicted all abnormalities constituting MetS in the present study, and we considered it pathologically justifiable for the UHDP was generated to explain the baseline WC and FBG. The reason for the HDP not being related to the elevated FBG and abdominal obesity can be partially explained by possible involvement of food items not being captured by the HDP on MetS development, but by the UHDP such as refined-carbohydrates and red/processed meat.

A systematic review of RCT showed that fish intake or n3 fatty acid supplements reduced WC compared with control [60]. Furthermore, the ingestion of seven nutrients abundant in a diet rich in fruits and vegetables, including soluble dietary fiber, Mg, K, vitamin K, folic acid, manganese and pantothenic acid, was significantly inversely correlated with the 2-year change in visceral fat [61]. In prospective research, whole grains, vegetables, fruits [62], legumes, and fish consumption were associated with reduction or a small increase in WC based on systematic review and meta-analysis [63].

Numerous systematic review and meta-analyses of prospective studies have reported food groups or nutrients in relationship with the incidence of type 2 diabetes (T2DM). The meta-evidence showed substantial beneficial effects in whole grain, cereal fiber and dairy [64,65] and moderate [66,67,68] or weak benefits [69] in fruits and/or vegetables. Dietary intakes of tofu, soy protein and soy isoflavones, but not total legumes or total soy, are inversely associated with incident T2DM [70]. In addition, a higher intake of Mg, which is most abundant in seeds and nuts, legumes and whole grains, was also found to be associated with the lower risk of T2DM [71,72]. Regarding n3 fatty acid consumption on the development of T2DM, an inverse association was reported in Asian populations [73,74,75,76], but a positive association was found in the Western population [73,74,75,76]. On the other hand, a meta-analysis of RCT did not support the idea that there are either the major harms or benefits from n3 fatty acid supplementation on insulin sensitivity compared to a placebo [77].

The magnitudes of the association found for the HDP and UHDP related to the risk of abdominal obesity and high FBG were different in the present study, which may emphasize the impact of additional food items identified by the UHDP. Although the UHDP score was found to be mildly correlated with total carbohydrate and protein compared to HDP, rice and noodles, most of which are refined grain products, and red and processed meats seemed to be important sources of UHDP, whereas fruits, starchy vegetables, soy products, fish and seafood may be substantial contributors to carbohydrate and protein in HDP. Indeed, the adverse effect of refined grain, red meat and sugar-sweetened beverages on weight gain or abdominal obesity was reported in a systematic review and meta-analysis of prospective studies [63]. Similarly, red and processed meat intake was found to be directly associated with higher BMI and WC according to a systematic review and a meta-analysis of observational studies [78]. In addition, recent systematic reviews and meta-analyses of RCT [79] or prospective studies [64,65,80] have elucidated that high consumption of red and processed meat [79] as well as diets with high glycemic-index and glycemic-load [79,81], such as those featuring high consumption of refined grains, sugar or artificial sugar-sweetened beverages [79], significantly increased the risk of T2DM. Besides the direct harms, these types of foods, in addition to pickles, may increase MetS risk by displacing other healthier foods, as observed in the present study.

Positive associations of UHDP with abdominal obesity were found in both men and women. However, the association in women was not as strong as that in men and was not statistically significant (*p*-interaction < 0.05). The weak association detected among women seems not likely due to the small number of women who developed abdominal obesity, since its incidence rate was triple as high for women as for men (175 incidence/605 women vs. 182 incidence/1933 men) in the present study, whereas those of hypertension, elevated TG and high FBG were twice as high for men as for women. Whether the discrepant magnitude of association was due to random variation needs further prospective studies with a large and identical sample size between men and women to confirm.

The strength of our study includes the prospective design, comprehensive valid food questionnaire and similar socio-economic backgrounds of the participants that are likely to preclude residual confounding. Our study also has several potential limitations. First, the dietary intakes were self-reported and measured only at baseline recalling for the past year; thus, the response error and changes in dietary intakes over time would have biased the results towards null. The RRR-derived dietary patterns may be population specific and lack reproducibility [82]. However, the derived dietary patterns share several features with existing Healthy/Unhealthy dietary patterns. Since the participants of the present study were exclusively government employees of Japan, the findings may not be readily generalizable to the community or other ethnic populations.

## 5. Conclusions

We generated healthy and unhealthy dietary patterns by applying RRR for predicting hypothesized-mediators of MetS: putatively beneficial nutrients and MetS components. Both dietary patterns showed dose-response associations with the incidence of MetS, while UHDP intake consistently predicted all abnormalities constituting MetS. Our results suggest that health education and practice that emphasize the merit of reducing the consumption of pickles, low-quality carbohydrates and red and processed meats which would consequently facilitate the intake of healthy diet and thus prevent MetS more efficiently.

## Figures and Tables

**Table 1:** Factor loadings and Pearson correlations between dietary pattern scores and consumption of food group, nutrient intakes, baseline waist circumference and fasting blood glucose for the Aichi Workers’ Cohort (*n* = 2944), 2013.

Healthy Dietary Pattern (HDP) ^†^			Unhealthy Dietary Pattern (UHDP) ^†^		
	Factor Loading	Pearson Correlation ^‡^		Factor Loading	Pearson Correlation ^‡^
Food Groups			Food Groups		
Vegetables, not specified	0.33	0.78 **	Tomatoes	−0.27	−0.34 **
Green vegetables, not specified	0.31	0.72 **	Bread	−0.25	−0.29 **
Green leafy vegetables	0.29	0.68 **	Dark-yellow vegetables	−0.23	−0.28 **
Dark-yellow vegetables	0.27	0.62 **	Sweeteners	−0.23	−0.27 **
Tomatoes	0.26	0.61 **	Green leafy vegetables	−0.21	−0.26 **
Cooking oil	0.26	0.60 **	Green vegetables, not specified	−0.18	−0.22 **
Other fruits	0.25	0.58 **	Millet	−0.17	−0.20 **
Mushroom	0.24	0.56 **	Mushroom	−0.14	−0.17 **
Potatoes	0.24	0.55 **	Vegetables, not specified	−0.11	−0.14 **
Soy products	0.23	0.54 **	Potatoes	−0.10	−0.13 **
Seaweed	0.22	0.52 **	Seaweed	−0.09	−0.12 **
Citrus fruits	0.22	0.51 **	Other fruits	−0.09	−0.11 **
Oily fish	0.15	0.35 **	Citrus fruits	−0.08	−0.10 **
Pickles	0.13	0.30 **	Confectioneries	−0.06	−0.08 **
Fish products	0.12	0.29 **	Whole-fat milk	−0.06	−0.08 **
Lean fish	0.12	0.28 **	Pasta	−0.04	−0.05 *
Seafood other than fish	0.11	0.27 **	Soy products	−0.04	−0.05 **
Dairy products	0.11	0.26 **	Oily fish	0.01	0.01
Salty fish	0.10	0.25 **	Corn oil	0.03	0.04 *
Millet	0.10	0.24 **	Dairy products	0.04	0.04 *
Nuts	0.09	0.21 **	Egg	0.05	0.06 **
Confectioneries	0.09	0.21 **	Chicken	0.06	0.06 **
Sweeteners	0.08	0.19 **	Cooking oil	0.06	0.07 **
Egg	0.06	0.13 **	Mayonnaise and margarine	0.06	0.08 **
Processed meats	0.04	0.10 **	Low-fat milk	0.09	0.11 **
Chicken	0.04	0.10 **	Lean fish	0.10	0.11 **
Bread	0.04	0.09 **	Miso soup	0.13	0.16 **
Mayonnaise and margarine	0.04	0.09 **	Salty fish	0.13	0.15 **
Miso soup	0.04	0.08 **	Nuts	0.14	0.17 **
Whole-fat milk	0.03	0.08 **	Fish products	0.15	0.17 **
Pasta	0.03	0.08 **	Pork	0.16	0.19 **
Low-fat milk	0.03	0.08 **	Processed meats	0.20	0.23 **
Noodles	0.02	0.05 **	Seafood other than fish	0.20	0.23 **
Corn oil	0.01	0.01	Liver	0.23	0.28 **
Liver	0.001	0.003	Beef	0.25	0.30 **
Pork	−0.002	−0.005	Noodles	0.27	0.32 **
Beef	−0.05	−0.12 **	Rice	0.28	0.34 **
Rice	−0.15	−0.36 **	Pickles	0.31	0.37 **
Response variables for HDP			Response variables for UHDP		
Total fiber		0.75 **	Fasting blood glucose		0.15 **
Vitamin C		0.68 **	Waist circumference		0.25 **
Vitamin E		0.66 **	Response variables for HDP		
Potassium		0.63 **	Total fiber		−0.37 **
Magnesium		0.58 **	Vitamin E		−0.28 **
β-carotene		0.57 **	β-carotene		−0.27 **
Omega-3 fatty acids		0.56 **	Potassium		−0.27 **
Protein		0.40 **	Vitamin C		−0.26 **
Ratio of protein to carbohydrates		0.34 **	Magnesium		−0.25 **
Carbohydrates		0.16 **	Omega-3 fatty acids		−0.08 **
Response variables for UHDP			Protein		−0.06 **
Fasting blood glucose		−0.03	Ratio of protein to carbohydrates		0.02
Waist circumference		−0.11 **	Carbohydrates		0.10 **

^†^ Dietary patterns were derived using reduced rank regression procedure. ^‡^ Pearson’s correlation was calculated using logarithmically transformed values. ** Significant at *p* < 0.01; * significant at *p* < 0.05.

**Table 2:** Baseline characteristics of the participants according to the quartiles of the Healthy Dietary Pattern for the Aichi Workers’ Cohort, 2013.

	Quartile of Healthy Dietary Pattern Score				*p*
	Q1, *n* = 910	Q2, *n* = 751	Q3, *n* = 646	Q4, *n* = 637	
Median (25%, 75%)	−1.56 (−2.34, −1.04)	−0.17 (−0.41, 0.08)	0.75 (0.53, 0.98)	1.83 (1.51, 2.41)	
Male, %	86.7	76.0	67.2	48.5	<0.0001
Age, year	46.4 (6.2)	46.6 (6.4)	47.1 (6.4)	46.8 (6.5)	0.22
Body mass index, kg/m^2^	22.7 (2.8)	22.5 (2.7)	22.1 (2.7)	22.0 (2.8)	<0.0001
Lower than university education, %	24.7	25.3	24.3	36.4	<0.0001
Non-clerical, %	42.3	47.8	46.9	48.0	0.06
Never smoker, %	57.6	64.7	65.9	74.9	<0.0001
Former smoker, %	23.9	22.4	21.8	17.7	
Current smoker, <20 cigarettes/day, %	9.9	6.8	7.9	5.3	
Current smoker, ≥20 cigarettes/day, %	8.7	6.1	4.3	2.0	
No change in eating habit, %	83.3	82.3	81.9	77.7	0.09
Changed within 1 year, %	4.0	5.1	2.9	5.2	
Changed in the past 1–2 years, %	6.6	5.9	7.0	9.9	
Changed in the past 3–5 years, %	6.2	6.8	8.2	7.2	
Alcohol consumption (g/day)	17.5 (23.5)	15.5 (21.8)	15.6 (21.3)	11.7 (18.1)	<0.0001
Total physical activity, MET-hours/day	34.9 (5.4)	35.4 (5.5)	35.9 (6.0)	36.0 (6.2)	0.0003
Total energy consumption, kcal/day	1869 (484)	2035 (509)	2104 (544)	2156 (555)	<0.0001
Waist circumference, cm	79.7 (8.4)	78.7 (7.9)	78.0 (7.9)	77.2 (8.1)	<0.0001
Systolic blood pressure, mmHg	121 (14)	119 (14)	117 (14)	115 (15)	<0.0001
Diastolic blood pressure, mmHg	75.8 (11.3)	74.9 (11.8)	73.2 (11.3)	71.5 (11.3)	<0.0001
Triglycerides, mg/dL	105 (75)	97.2 (58.9)	93.5 (59.6)	83.0 (46.7)	<0.0001
High-density lipoprotein cholesterol, mg/dL	62.4 (14.9)	63.1 (14.6)	65.1 (15.9)	67.8 (16.1)	<0.0001
Fasting blood glucose, mg/dL	91.7 (11.8)	91.1 (9.7)	90.6 (8.0)	90.7 (11.0)	0.13
Medication for hypertension, %	6.3	5.1	5.7	4.6	0.48
Medication for hyperglycemia, %	0.33	0.53	0.93	0.31	0.35
Medication for dyslipidemia, %	0	0	0	0	-

Data were means (standard deviation) or percentage. Differences were tested by using a generalized linear model.

**Table 3:** Baseline characteristics of the participants according to the quartiles of the Unhealthy Dietary Pattern for the Aichi Workers’ Cohort, 2013.

	Quartile of Unhealthy Dietary Pattern Score				*p*
	Q1, *n* = 639	Q2, *n* = 662	Q3, *n* = 746	Q4, *n* = 897	
Median (25%, 75%)	−1.16 (−1.52, −0.92)	−0.35 (−0.51, 0.22)	0.18 (0.03, 0.32)	0.90 (0.69, 1.26)	
Male, %	53.7	65.7	75.1	85.3	<0.0001
Age, year	45.1 (6.1)	46.3 (6.4)	47.0 (6.4)	47.9 (6.3)	<0.0001
Body mass index, kg/m^2^	21.5 (2.5)	22.1 (2.6)	22.4 (2.8)	23.1 (2.8)	<0.0001
Lower than university education, %	27.9	29.0	24.7	27.9	0.28
Non-clerical, %	46.5	45.8	46.5	45.3	0.95
Never smoker, %	76.8	69.2	65.0	53.4	<0.0001
Former smoker, %	16.4	18.9	21.3	27.9	
Current smoker, <20 cigarettes/day, %	4.5	7.7	8.2	9.5	
Current smoker, ≥20 cigarettes/day, %	2.2	4.2	5.5	9.3	
No change in eating habit, %	80.0	81.1	81.8	82.7	0.52
Changed within 1 year, %	5.5	4.2	3.0	4.6	
Changed in the past 1–2 years, %	6.9	7.4	7.9	6.7	
Changed in the past 3–5 years, %	7.7	7.3	7.4	6.0	
Alcohol consumption (g/day)	8.6 (13.9)	14.2 (20.5)	16.1 (21.7)	20.3 (25.2)	<0.0001
Total physical activity, MET-hours/day	35.6 (6.0)	35.7 (5.9)	35.4 (5.6)	35.3 (5.6)	0.54
Total energy consumption, kcal/day	1786 (464)	1969 (502)	2079 (523)	2192 (537)	<0.0001
Waist circumference, cm	75.9 (7.8)	77.6 (7.4)	78.8 (8.0)	81.0 (8.3)	<0.0001
Systolic blood pressure, mmHg	114 (14)	118 (15)	119 (14)	121 (14)	<0.0001
Diastolic blood pressure, mmHg	70.9 (11.0)	73.3 (11.7)	74.7 (11.4)	76.4 (11.4)	<0.0001
Triglycerides, mg/dL	81.4 (45.7)	89.8 (57.5)	98.2 (63.8)	108 (72)	<0.0001
High-density lipoprotein cholesterol, mg/dL	67.2 (15.8)	65.6 (16.2)	63.6 (15.2)	61.9 (14.4)	<0.0001
Fasting blood glucose, mg/dL	89.4 (8.1)	90.6 (9.8)	90.9 (9.6)	92.9 (12.4)	<0.0001
Medication of hypertension, %	4.5	4.8	5.5	6.6	0.29
Medication of hyperglycemia, %	0.47	0.60	0.13	0.78	0.32
Medication of dyslipidemia, %	0	0	0	0	-

Data were means (standard deviation) or percentage. Differences were tested by generalized linear model.

**Table 4:** Hazard ratios and 95% confidence intervals for the incidence of metabolic syndrome and its components according to the quartiles of the Healthy Dietary Pattern score for the Aichi Workers’ Cohort, 2013–2018.

	Q1	Q2		Q3		Q4		Trend-*p*	*P*-Interaction for Sex
		HR (95% CI)	*p*	HR (95% CI)	*p*	HR (95% CI)	*p*		
Metabolic syndrome incidence									
No. of subjects	910	751		646		637			
No. of incidence	133	101		74		66			
Person-years	3614	3112		2666		2589			
Crude incidence rate *	36.8	32.5		27.8		25.5			
Model 1	1	0.81 (0.62–1.05)	0.11	0.65 (0.49–0.88)	0.005	0.60 (0.44–0.82)	0.001	0.0004	0.31
Model 2	1	0.84 (0.64–1.09)	0.18	0.68 (0.50–0.91)	0.009	0.65 (0.48–0.90)	0.009	0.002	0.40
Individual diagnostic criteria for metabolic syndrome									
Hypertension									
No. of subjects	614	524		470		500			
No. of incidence	254	184		166		138			
Model 1	1	0.79 (0.66–0.94)	0.01	0.80 (0.66–0.97)	0.02	0.69 (0.55–0.85)	0.001	0.001	0.70
Model 2	1	0.81 (0.67–0.97)	0.02	0.82 (0.68–1.00)	0.049	0.71 (0.57–0.88)	0.002	0.002	0.60
Elevated triglyceride									
No. of subjects	778	664		578		602			
No. of incidence	173	122		100		91			
Model 1	1	0.81 (0.65–1.02)	0.07	0.80 (0.62–1.02)	0.08	0.79 (0.61–1.03)	0.08	0.051	0.18
Model 2	1	0.81 (0.66–1.05)	0.12	0.81 (0.63–1.04)	0.09	0.81 (0.63–1.06)	0.13	0.08	0.17
Decreased high-density lipoprotein cholesterol									
No. of subjects	892	736		625		616			
No. of cases	87	72		58		49			
Model 1	1	0.92 (0.67–1.27)	0.62	0.84 (0.60–1.19)	0.33	0.70 (0.49–1.02)	0.06	0.06	0.22
Model 2	1	0.96 (0.71–1.31)	0.81	0.86 (0.61–1.20)	0.37	0.73 (0.50–1.05)	0.09	0.08	0.23
Abdominal obesity									
No. of subjects	805	646		567		520			
No. of incidence	87	89		88		93			
Model 1	1	1.07 (0.80–1.43)	0.64	1.07 (0.79–1.44)	0.67	1.02 (0.75–1.39)	0.92	0.91	0.51
Model 2	1	1.10 (0.82–1.47)	0.54	1.11 (0.82–1.51)	0.48	1.04 (0.76–1.43)	0.79	0.75	0.42
Elevated fasting blood glucose									
No. of subjects	801	671		589		573			
No. of incidence	141	125		103		78			
Model 1	1	1.00 (0.79–1.27)	0.99	0.92 (0.71–1.18)	0.49	0.80 (0.59–1.07)	0.13	0.13	0.22
Model 2	1	1.02 (0.80–1.29)	0.88	0.93 (0.72–1.21)	0.58	0.82 (0.61–1.11)	0.19	0.20	0.24

*: 1000 person-years. Model 1 was adjusted for age (continuous), sex and total energy consumption (continuous). Model 2 was further adjusted for total physical activity (sex-specific quartiles), smoking status (never; former; current, <20 cigarettes/day; and current, ≥20 cigarettes/day), education (lower than university education and others), work (clerical and non-clerical), change in eating habits (no change, changed within 1 year, changed in the past 1–2 years and changed in the past 3–5 years) and medication for dyslipidemia (yes and no), antihypertension (yes and no) and hyperglycemia (yes and no).

**Table 5:** Hazard ratios and 95% confidence intervals for the incidence of metabolic syndrome and its components according to the quartiles of the Unhealthy Dietary Pattern score for the Aichi Workers’ Cohort, 2013–2018.

	Q1	Q2		Q3		Q4		Trend-*p*	*P*-Interaction for Sex
		HR (95% CI)	*p*	HR (95% CI)	*p*	HR (95% CI)	*p*		
Metabolic syndrome incidence									
No. of subjects	639	662		746		897			
No. of incidence	42	68		101		163			
Person-years	2750	2761		3008		3462			
Crude incidence rate *	15.3	24.6		33.6		47.1			
Model 1	1	1.49 (1.02–2.18)	0.04	1.96 (1.37–2.81)	0.0003	2.62 (1.85–3.70)	<0.0001	<0.0001	0.19
Model 2	1	1.48 (1.01–2.16)	0.04	2.01 (1.40–2.88)	0.0001	2.51 (1.77–3.54)	<0.0001	<0.0001	0.24
Individual diagnostic criteria for metabolic syndrome									
Hypertension									
No. of subjects	509	477		543		579			
No. of incidence	129	150		210		253			
Model 1	1	1.13 (0.91–1.42)	0.27	1.32 (1.07–1.64)	0.01	1.47 (1.19–1.82)	0.0004	0.0001	0.72
Model 2	1	1.13 (0.90–1.41)	0.29	1.29 (1.04–1.60)	0.02	1.39 (1.12–1.72)	0.003	0.001	0.72
Elevated triglyceride									
No. of subjects	603	606		657		756			
No. of incidence	75	114		123		174			
Model 1	1	1.44 (1.08–1.92)	0.01	1.37 (1.03–1.83)	0.03	1.64 (1.24–2.17)	0.001	0.001	0.90
Model 2	1	1.42 (1.07–1.90)	0.02	1.37 (1.03–1.83)	0.03	1.60 (1.21–2.12)	0.001	0.002	0.83
Decreased high-density lipoprotein cholesterol									
No. of subjects	620	641		733		875			
No. of cases	36	57		81		92			
Model 1	1	1.58 (1.04–2.41)	0.03	2.03 (1.37–3.01)	0.0005	1.98 (1.33–2.96)	0.001	0.0004	0.76
Model 2	1	1.58 (1.04–2.40)	0.03	2.06 (1.39–3.06)	0.0003	1.94 (1.30–2.88)	0.001	0.001	0.69
Abdominal obesity									
No. of subjects	547	581		637		773			
No. of incidence	67	83		90		117			
Model 1	1	1.40 (1.02–1.91)	0.04	1.61 (1.18–2.21)	0.003	2.05 (1.51–2.78)	<0.0001	<0.0001	0.02
Model 2	1	1.37 (1.00–1.86)	0.0496	1.61 (1.17–2.21)	0.003	1.93 (1.42–2.62)	<0.0001	<0.0001	0.01
Elevated fasting blood glucose									
No. of subjects	599	601		667		767			
No. of incidence	69	99		117		162			
Model 1	1	1.31 (0.97–1.77)	0.08	1.32 (0.99–1.78)	0.06	1.52 (1.14–2.03)	0.004	0.006	0.83
Model 2	1	1.30 (0.96–1.76)	0.09	1.31 (0.98–1.76)	0.07	1.46 (1.09–1.97)	0.01	0.01	0.95

*: 1000 person-years. Model 1 was adjusted for age (continuous), sex and total energy consumption (continuous). Model 2 was further adjusted for total physical activity (sex-specific quartiles), smoking status (never; former; current, <20 cigarettes/day; and current, ≥20 cigarettes/day), education (lower than university education, and others), work (clerical and non-clerical), change in eating habits (no change, changed within 1 year, changed in the past 1–2 years and changed in the past 3–5 years) and medication for dyslipidemia (yes and no), antihypertension (yes and no) and hyperglycemia (yes and no).

## Data Availability

Raw data supporting reported results of this study are human research participant data and are not publicly available, but could be made available upon justified requests and after appropriate procedures, including approval from the institutional ethics review committee.
